# The Effect of the Loading Frequency on the Dynamic Bending Strength of Spruce Wood

**DOI:** 10.3390/ma17184662

**Published:** 2024-09-23

**Authors:** Enej Lipovec Zupanc, Miha Humar, Gorazd Fajdiga

**Affiliations:** Department of Wood Science and Technology, Biotechnical Faculty, University of Ljubljana, Jamnikarjeva 101, 1000 Ljubljana, Slovenia; enej@sloform.eu (E.L.Z.); miha.humar@bf.uni-lj.si (M.H.)

**Keywords:** dynamic strength, frequency, fatigue, spruce, wood

## Abstract

Wood is increasingly being used in construction as an alternative to steel and concrete. As wood is an inhomogeneous material, this has a strong effect on its static and dynamic properties. When timber is used as a load-bearing component, there is a possibility that it will be exposed to unfavourable weather conditions (wind) or dynamic environments (vibrations), leading to fatigue of the material. In this article, the effects of load frequency and load magnitude on the durability of Norway spruce wood (*Picea abies*) were investigated. The frequencies of 5 and 10 Hz were compared at three load levels of 70%, 80% and 90% of the static breaking force. The research results show that the load magnitude has a major influence on the dynamic strength at the same fatigue frequency. Each increase in load means a lower dynamic strength of the spruce, which is reflected in the load cycles achieved. In addition, the dynamic properties of spruce wood deteriorate with an increasing loading frequency, which is more pronounced at higher loading forces. These test results are the basis for determining the Wöhler curve, which is required as input data for the material properties in numerical calculations to determine the durability of the material.

## 1. Introduction

In its unaltered form, wood is a unique, economically significant, technical natural material [1]. As a material, wood is characterised by excellent mechanical performance and competitiveness compared to other known building materials (concrete, iron, reinforced concrete, steel, etc.). The most important advantages of wood are its renewability, ease of processing and the good balance between mass and mechanical properties [2].

Wood was undoubtedly one of the first materials to be used for construction purposes [3]. The oldest shelters in human history were built of wood several thousand years ago. Wood was used because it is much easier to work with than other traditional materials such as stone. In some parts of the world, such as North America, Scandinavia and Australia, it is still the most commonly used building material for residential purposes. In southern Europe, on the other hand, wood is used less frequently due to termite infestation and other building traditions. However, a renaissance in the gutting of wooden buildings can be observed. It should be borne in mind that modern construction would not be possible without the use of modern wood-based materials such as glulam, cross-laminated timber or laminated veneer lumber. In the construction industry, great importance is attached to the mechanical properties of wood, which are a very important criterion for the use of wood in construction [4].

The static mechanical properties (bending strength, tensile strength, compressive strength, etc.) are among the mechanical properties that are essential for the construction of load-bearing structures [5]. In modern design guidelines for construction, the dynamic mechanical properties of wood used for construction are increasingly taken into account. Timber components are often subjected to quasi-static loads in construction [6]. The Eurocode suggests that the service life of permanent structures, such as tall buildings and bridges, should be 100 years or more. With such a long service life, fatigue becomes increasingly important. There are numerous studies investigating different materials and manufacturing processes to develop advanced wood products with a higher load-bearing capacity and stiffness [6,7,8,9].

The mechanical properties of wood as a building material are determined from the point of view of linear elasticity in areas with low humidity and temperature and at low stresses [10]. Wooden structures are often exposed to increased moisture load. If the moisture period is short, it affects the viscoelastic properties of the wood. Longer periods of moisture are required for the decomposition of Norway spruce wood. The first signs of fungal degradation on the wood can be expected after 325 days under favourable conditions. Wood is considered a viscoelastic material, which means that time-dependent deformations occur under permanent loads. The mechanical properties of wood are influenced by inherent properties such as density, orientation, wood fibre orientation, microfiber angle and anomalies due to growth characteristics [6,11]. In addition, catastrophic events such as windthrow or ice storms can also influence the mechanical properties. Therefore, wood from sanitary timber harvesting cannot be used for all applications.

Static loads do not change in magnitude and direction with time. When loads occur, they increase only slowly to their nominal value. Under normal static loads, the material expands (tensile stresses) or shrinks (compressive stresses), resulting in a normal specific deformation (ε) [12].

If components are frequently exposed to the weather (wind) or are used in a dynamic environment (shocks, vibrations, repeated mechanical loads), material fatigue occurs, which can also lead to breakage [13]. Dynamic loads often lead to permanent deformation, cracks and fractures in the material at lower stresses than static loads [7,14,15,16,17]. One of the best-known material failures due to fatigue is the accident of the first jet aeroplane of the airline de Havilland Comet. 

The static strength and fatigue strength of wood must therefore be known for the design of components. One of the first studies on the fatigue of wood was carried out by Wood [18], who presented the so-called "Madison curve" as the relationship between stress and load duration. This concept helps wood scientists and engineers predict the behaviour of wood materials under various loading conditions, which is critical for construction, furniture manufacturing and other wood product applications. Understanding the Madison curve is crucial in industries such as construction, as the strength of wood needs to be accurately predicted in different environments. In recent years, much research has been carried out to explain the fatigue phenomena of wood [2,16,17,19,20,21,22].

The strength and dynamic properties of materials are determined experimentally [23]. The test specimens are subjected to a periodically varying load (dynamic load factor R) until cracks or fractures occur. The number of load cycles N at which the test specimens break is measured.

The tests carried out on Norway spruce (*Picea abies*) samples showed an increase in static bending strength and a decrease in fatigue strength due to the wood’s density. The influence of processing is insignificant for static strength, but obvious for dynamic loading. It has been shown that radial loads cause greater damage than tangential loads, which is mainly due to the effect of layering. 

The occurrence of damage in wood depends on the loading cycle performed and the cycle shape, which determine key factors such as the loading rate and the dwell time at peak load [2].

The characterization of the static properties of the material has already been published by several authors [23,24,25,26,27]. The content of this article is part of a broader investigation of the influence of dynamic loads on the fatigue of spruce wood and is a continuation of research previously published in the journal *Materials* [28]. In this paper, the focus is on evaluating the influence of loading frequency on the dynamic response of spruce wood. With the present study, we aim to confirm the hypothesis that the dynamic strength of spruce wood decreases with increasing loading frequency. 

Based on the experimental results of this study, the Wöhler curve can be determined using different models [2,15,24,25,26,27,28]. We use the model developed by us, which is described in detail in reference [28] and also takes into account the greater scatter of the results, which occurs very frequently with wood heterogeneity. The Wöhler curves are required as input for numerical models for calculating product service life or durability [2,15].

## 2. Materials and Methods

The influence of the loading frequency on the dynamic strength of the wood was investigated on air-dried spruce wood. The wood samples showed no growth anomalies such as cracks, reaction wood or signs of decay, as these could have a decisive influence on the test results. Spruce (*Picea abies* (L.) Karst.) has a characteristically soft and resilient wood and is one of the most important wood species for construction applications. Spruce wood is an important import source for the construction, furniture, pulp and paper industries in central and northern Europe. One of the niche applications with high added value is the manufacture of musical instruments. The dry density of spruce wood is between 290 and 380 kg/m^3^ for earlywood and between 820 and 910 kg/m^3^ for latewood [2,15]. The samples with a cross-section of 10 mm × 10 mm were made from a homogeneous radial board of spruce wood. The wood was sourced from Slovenian forests.

**Figure 1:**
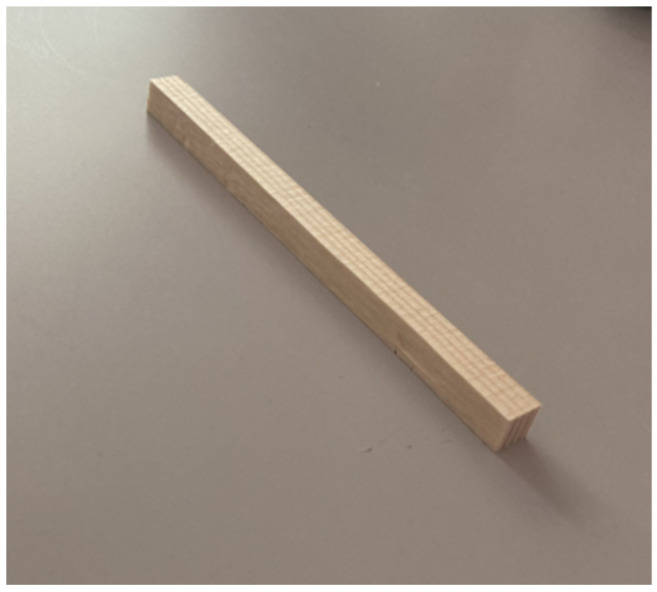
Final specimen dimensions: 10 mm × 10 mm × 150 mm.

Due to dimensional instability, which is closely related to variations in wood moisture content, the resulting specimens were conditioned under laboratory conditions (T = 20 °C and φ = 65%), as prescribed in the ASTM D 143-94 standard [29]. Finally, the battens were machined to the final dimensions of the test specimens, 10 mm × 10 mm, and sawn to a length of 150 mm (Figure 1). The density of the specimens was determined by measuring the mass and dimensions of the specimens and by measuring the moisture content according to the standard EN 13183-1:2003 [30]. Due to the variability of the wood, it is important that the number of test specimens is sufficient [31].

Small, clean specimens are used to determine the strength of the wood, as prescribed in the relevant standards [30,31]. The specimens were selected by experienced personnel familiar with a variety of growth anomalies and decay patterns. The selection was made by visual inspection, which is the most accurate and sensitive method. Prior to testing, the specimens were conditioned in a normal climate (20 °C and 65% humidity).

The static bending test is mainly used for the mechanical classification of wood; therefore, this method is considered the most commonly used to determine the strength properties of wood. To determine the static bending strength, 10 random specimens were selected and subjected to a bending test according to EN 408 (2003), Figure 2 [32].

**Figure 3:**
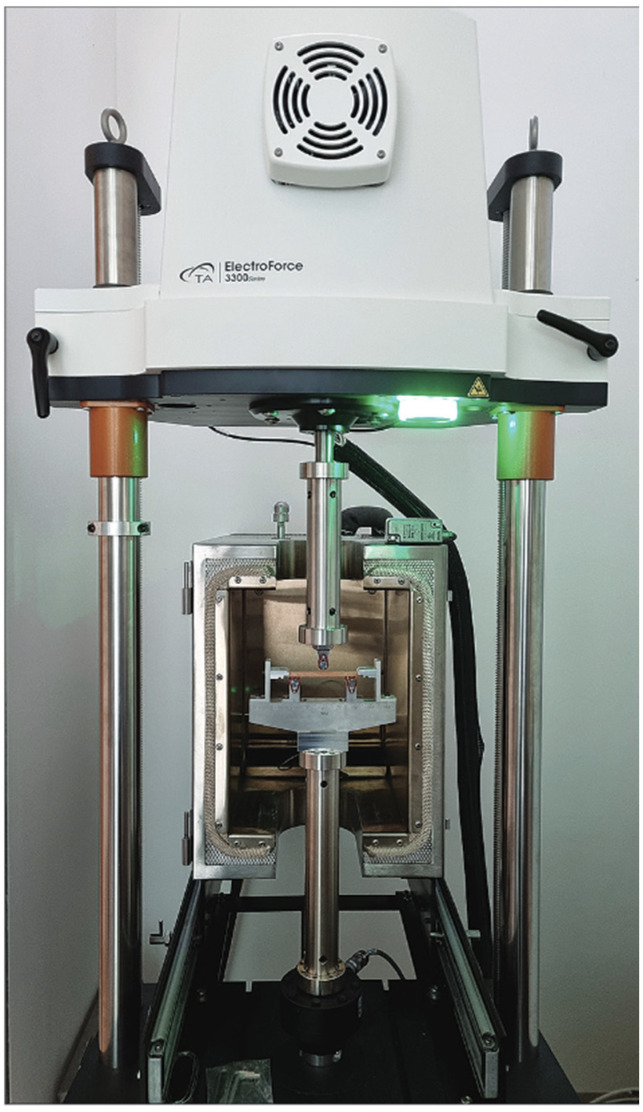
DMA Electroforce 3310 Series III commercial test bench.

To determine the dynamic bending strength and obtain the durability curve of the materials, the specimens must be tested on fatigue test rigs. Such tests require very precise test rigs with all measuring elements and the design itself with all components. Quality and precision in the design and assembly of the test rig are very important. We have developed special devices for material fatigue tests for this purpose. These are only intended for testing larger samples at lower loading frequencies. To investigate the effect of frequency on durability, we used the commercially available DMA Electroforce 3310 Series III test rig (Figure 3) in this study, which provides a sufficiently high fatigue frequency to obtain results within a reasonable time. The device we used for the fatigue tests can achieve a maximum compressive force of 1000 N at a frequency of 10^−5^ Hz to 100 Hz. It is also possible to adjust the climatic conditions of the test, as the temperature of the ambient air in the chamber can be changed during the fatigue itself.

During the fatigue process, the load force and deformation data are transferred to the computer for final analysis. In our case, we recorded the data four times per second. If the device detects a fracture in the sample, the test is automatically terminated.

The number of samples used and the distribution of samples among the different test groups are shown in Table 1. The choice of the percentage value of the static breaking force was based on the tests already carried out and the experience gained from modelling the Wöhler curve with our model [11,28].

## 3. Results

The average measured density of the test specimens used at a fatigue frequency of 5 Hz was 0.457 g/cm^3^, which according to Wangaard [15] corresponds to the density range for spruce wood. The samples used to determine the fatigue force at a frequency of 5 Hz withstood an average static force of 551 N in the static bending tests (Table 2). 

Based on the measurements taken, the average measured density of the specimens used at a fatigue frequency of 10 Hz was 0.478 g/cm^3^. The samples used to determine the fatigue force at a frequency of 10 Hz withstood an average static force of 591 N during the static bending tests (Table 2). 

### 3.1. Fatigue at a Frequency of 5 Hz

At 70% of an average static breaking force, the test specimens reached an average of 2,040,364 cycles, and the fatigue force amounted to 385 N (Table 2). The modulus of elasticity (E) was on average 9.711 MPa and the density 0.451 g/cm^3^.

A good half (61%) remained intact and reached 2,500,000 cycles, the upper limit we chose so as not to run the tests for too long. As many as 11% of the specimens underwent fewer than 1,000,000 fatigue cycles, while 22% of the specimens reached between 1,000,000 and 2,000,000 cycles. The remaining 6% of the specimens reached between 2,000,000 and 2,500,000 cycles.

Compared to the average fatigue density of the specimens at a frequency of 5 Hz, the group with an average static breaking force percentage of 70 has a slightly lower density, and consequently, the modulus of elasticity is lower. The number of load cycles achieved is to be expected as the force level is the lowest. Compared to the loading frequency of 10 Hz, the specimens fatigued at 10 Hz achieved a higher number of load cycles, which is due to the slightly higher density.

At 80% of an average static breaking force, the specimens achieved an average of 743,615 cycles and the fatigue force averaged 441 N (Table 2). The modulus of elasticity (E) averaged 9.787 MPa and the specimen density was 0.454 g/cm^3^. The percentage of intact specimens was 12%, which means that they reached 2,500,000 cycles. Seventy-one percent of the specimens reached fewer than 1,000,000 cycles and 50 did not reach 100,000 cycles. A total of 6% of the specimens achieved between 1,000,000 and 2,000,000 load cycles.

At 90% of the required force, the specimens reached an average of 296,451 cycles; the fatigue force amounted to 496 N (Table 2). The modulus of elasticity (E) averaged 10.163 MPa and the sample density was 0.463 g/cm^3^.

Only 5% of the specimens remained intact or reached 2,500,000 cycles. On average, the specimens withstood 296,451 cycles. Most specimens (74%) did not reach 100,000 cycles, and a third (35%) did not reach 1000 fatigue cycles. A total of 16% of the specimens withstood between 100,000 and 1,000,000 cycles, 5% between 1,000,000 and 2,000,000 cycles and the same number between 2,000,000 and 2,500,000 cycles (Table 3).

As with 70 and 80 percent of an average static breaking force, there are also large differences in the load cycles achieved with 90 percent of an average breaking force. At the same time, we have a specimen that has withstood all 2,500,000 load cycles and, on the other hand, a specimen that has only withstood 433 load cycles under the same conditions.

### 3.2. Fatigue Frequency Test Group 10 Hz

At 70% of an average static breaking force, the specimens reached an average of 2,397,888 cycles, and the fatigue force amounted to 414 N (Table 2). The modulus of elasticity (E) averaged 11.105 MPa and the specimen density was 0.474 g/cm^3^.

A total of 90% of the specimens remained intact for up to 2,500,000 cycles. A total of 10% of the test specimens achieved between 1,000,000 and 2,000,000 cycles.

The lowest number of cycles (1,478,876) was achieved by the test specimen with a density of 0.472 g/cm^3^, which is almost 40% less than the other test specimens. The maximum difference in density between the test specimens that underwent all 2,500,000 cycles was 10%. The specimen with the lowest density (0.450 g/cm^3^) achieved the same number of cycles as the specimen with the highest density (0.496 g/cm^3^).

At 80% of an average static breaking force, the specimens reached an average of 557,281 cycles, and the fatigue force amounted to 473 N (Table 2). The modulus of elasticity (E) averaged 11.067 MPa and the specimen density was 0.480 g/cm^3^.

The percentage of intact specimens was 0%; i.e., no specimens reached 2,500,000 cycles. The maximum number of load cycles was achieved by one specimen, which reached 2,485,885 cycles. A total of 90% of the test specimens reached fewer than 1,000,000 cycles and 22% did not even reach 100,000 load cycles.

The lowest number of cycles (12,495) was achieved by the specimen with a density of 0.485 g/cm^3^, which is about 199 times less than the specimen with a density of 0.483 g/cm^3^, which achieved the highest number of load cycles (2,485,885). Within the sample group, the sample with the lowest density (0.457 g/cm^3^) has a density 8% lower than the sample with the highest density, 0.492 g/cm^3^.

At 90% of an average static breaking force, the test specimens achieved an average of 22,099 cycles with a fatigue force of 532 N (Table 2). The modulus of elasticity (E) averaged 11.128 MPa and the density of a specimen was 0.480 g/cm^3^.

None of the specimens reached all 2,500,000 load cycles, and there was no specimen that reached 1,000,000 load cycles. The specimen with a density of 0.491 g/cm^3^ achieved the highest number of load cycles, namely 125,646 load cycles (Table 4).

## 4. Discussion

**Figure 4:**
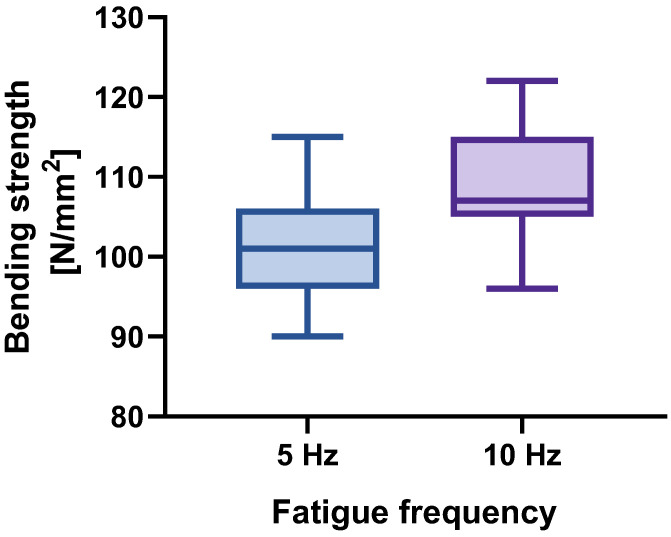
Static bending strength of specimens subjected to fatigue at 5 Hz and 10 Hz.

The fatigue force was determined for both groups by static fracture tests. We calculated the static bending strength of the specimens from the static breaking force data. Figure 4 shows the achieved bending strength of the specimens for the load group at frequencies of 5 Hz and 10 Hz. 

**Figure 5:**
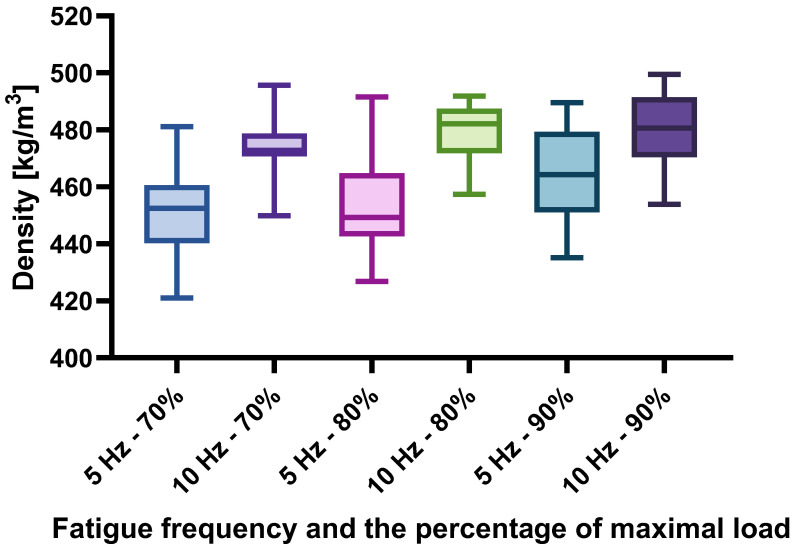
Density of specimens.

Figure 4 shows that the group of specimens designed to determine the fatigue force at a load frequency of 5 Hz achieved a lower static bending strength than the group of specimens designed to determine the fatigue force at a frequency of 10 Hz. The differences are due to material differences, with density having the greatest influence on static bending strength and other mechanical properties. This phenomenon is well known. The density of conifers is mainly related to the ring width. Since the ring widths of conifers depend on the annual climate, the ring widths cannot be the same every year. Above all, the proportion of latewood influences the density and the associated mechanical properties. The volume of latewood in the ring width is almost constant. If the rings are narrow, they have a relatively higher proportion of latewood, which leads to in better mechanical properties. We have tried to minimise this effect by carefully selecting the samples, but the density effect cannot be completely eliminated. This is confirmed by the analysis of the densities shown in Figure 5. Due to the differences in the bending strength achieved, the fatigue forces also differ between the groups of test subjects. Therefore, it is crucial to determine the magnitude of the force, expressed as a percentage based on the measured bending strength for each group of test specimens (in our case we used 70%, 80% and 90% of an average static breaking force for dynamic load).

At a fatigue frequency of 5 Hz, the expected differences between the different load levels can be observed. The number of load cycles achieved decreases with increasing load force, which is confirmed by [33]. The higher the force, the higher the stresses and the faster microcracks occur, which contribute significantly to the failure of the specimen. The reduction in load cycles with the increase in force can be observed both at the frequency of 5 Hz and at the fatigue frequency of 10 Hz. Fatigue force has a significant influence on dynamic strength, which is confirmed by the difference in load cycles, which is up to 274% between 70% and 80% static force at a frequency of 5 Hz, up to a 250% difference in the cycles achieved between 80% and 90% of an average static breaking force and up to 688% between 70% and 90% of an average static breaking force. At a frequency of 5 Hz, an increase in fatigue force of 10% therefore leads to a 2.6-fold reduction in the number of load cycles.

At a fatigue frequency of 10 Hz, the number of load cycles achieved decreases with each increase in load force. Compared to the fatigue frequency of 5 Hz, the differences in the load cycles between the individual load forces are greater. For example, between 70% and 80% of an average static force, the difference in the load cycles achieved is 430%. Between 80% and 90% of an average static breaking force, the difference is as much as 2.521%. The difference between 70% and 90% of an average static breaking force is 108 times greater at a fatigue frequency of 10 Hz. The results show that the differences are greater at a higher frequency. 

Karr et al. [34] came to a different conclusion, namely that there are no differences in bending fatigue between 50 Hz and 20 kHz, or that these are negligible in birch wood. Schönbauer [15] and his colleagues found the same, when it came to maple wood.

For the same order of magnitude of loading frequencies used in the tests (up to 10 Hz), Clorious [35] came to the same conclusions and demonstrated the influence of frequency on the dynamic strength of spruce wood at frequencies between 0.1 and 10 Hz.

On average, the test subjects achieved 1,018,529 load cycles at a loading frequency of 5 Hz, which is 26,106 load cycles more than the test subjects achieved on average at a loading frequency of 10 Hz. 

Despite the slightly higher density of the specimens that fatigued at 10 Hz, a lower average number of load cycles was achieved at the lower frequency for all force magnitude classes. If the specimens had the same density at both load frequencies, the difference in the number of load cycles would probably be even more pronounced.

When comparing the results in terms of the number of load cycles achieved between different load frequencies for the same force magnitude class, it can be seen that the number of load cycles generally decreases with increasing frequency. The difference becomes clearer with each increase in load force. What is interesting about the results is that the scatter of the results is significantly lower at a higher load frequency than at a lower one. It can be assumed that this is due to the difference between the material used for the test group at 5 Hz and 10 Hz.

**Figure 6:**
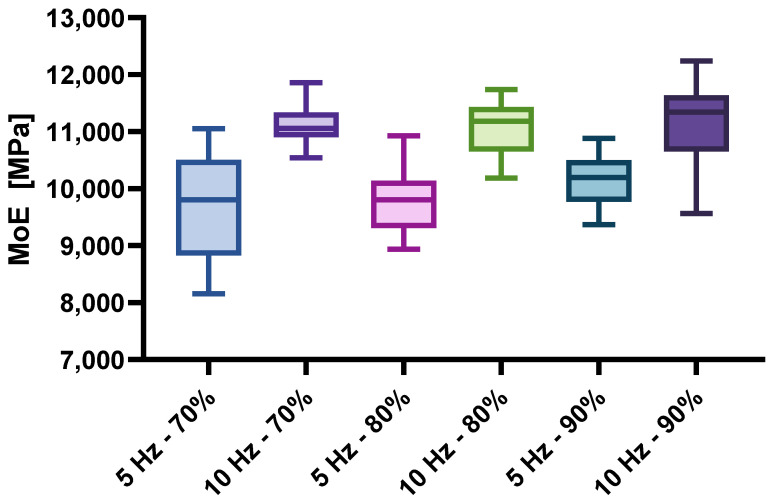
Modulus of elasticity of the specimens.

**Figure 7:**
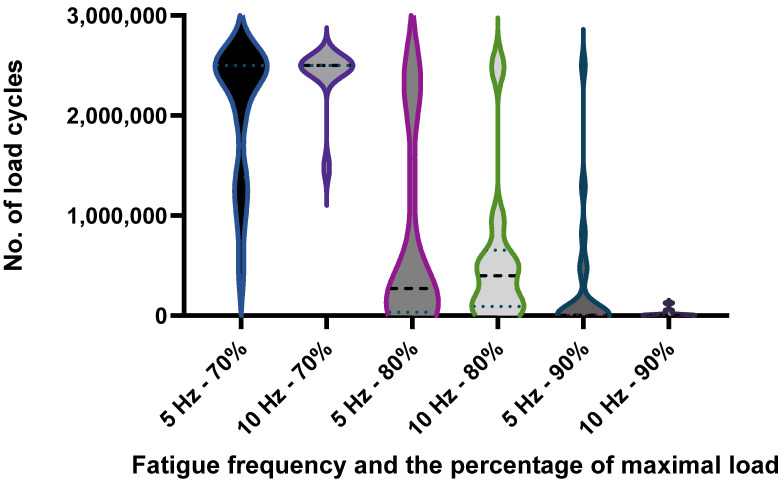
The number of load cycles of the specimens.

In the following figures, the results are shown separately according to the load frequency and the force percentage with which the test specimens were loaded. Figure 5 shows the density of the test specimens, Figure 6 the modulus of elasticity of the specimens and, finally, Figure 7 the number of load cycles achieved by the specimens within each test group.

As already suspected on the basis of the bending strength result that the reason for the differences lies in the density of the samples, this is confirmed here. It can be observed that the group of specimens loaded at 10 Hz achieved higher density values than the specimens subjected to a loading frequency of 5 Hz. As the density is closely related to the physical and mechanical properties of the wood, this is shown not only by the comparison of the bending strength, but also by the comparison of the modulus of elasticity between the individual sample groups, which is shown in Figure 6. Figure 5 and Figure 6 are very similar, which confirms the statement by Wangaard [15] that the density is closely correlated with the mechanical properties of the wood.

What interests us about the study is shown in Figure 7, where we compare the number of loading cycles between the different groups, which were divided according to the load frequency (5 and 10 Hz) and the magnitude class of the loading force (70%, 80% and 90% of an average static breaking force). It can be seen that the number of load cycles decreases with each increase in load force regardless of the load frequency used.

## 5. Conclusions

Based on the results, we can see that minor variations in density are not crucial for our study, as the differences in density do not contribute to major differences between the specimens. Variations in the density of solid wood are quite common, as many different factors in the structure of the wood occur in different proportions.

It has been found that the dynamic strength of spruce wood decreases with increasing loading frequency at higher loading forces. At higher fatigue forces, the deformation is greater, which leads to faster material degradation. At higher loading frequencies, the material cannot relax either, as it does not have enough time to react during the loading cycles. Based on the results, the hypotheses can be confirmed.

Fatigue tests have shown that the fatigue frequency has a significant influence, which is more pronounced at higher loads. With increasing load frequency, the number of load cycles achieved decreases significantly. It would be useful to compare a wider frequency range, as this would make it easier to predict what generally happens to the material during fatigue at different load frequencies.

The research results presented are used in the design of timber structures and in predicting the service life of existing spruce structures. The research results presented for spruce wood are used to determine the Wöhler curve, which is required as input data for the material properties in numerical calculations in order to simulate the wood fatigue process and predict the service life or dynamic strength of spruce wood with a certain probability. 

## Figures and Tables

**Figure 2:**
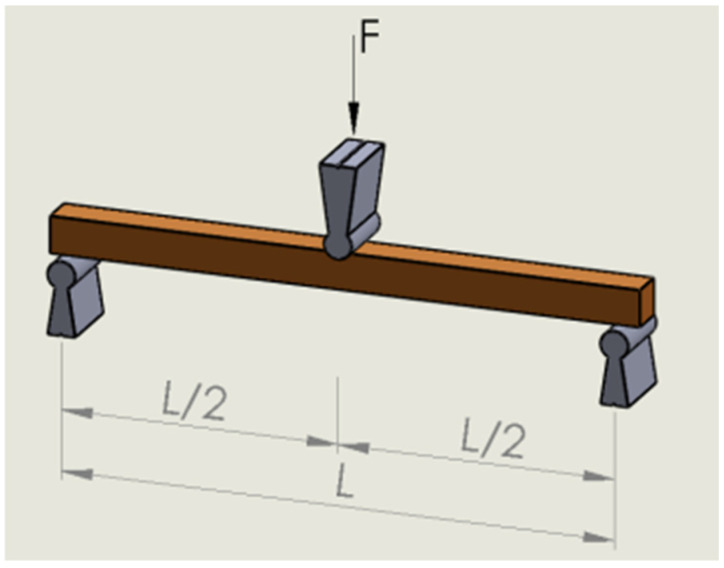
Demonstration of static bending strength test as prescribed in EN 408 (2003) [32].

**Table 1:** Display of the distribution of samples according to the parameters of frequency and the fraction of the static breaking force Fp.

Percentage of Static Breaking Force (%)	Number of Samples (Frequency 5 Hz)	Number of Samples (Frequency 10 Hz)
70	10	10
80	10	10
90	10	10

**Table 2:** Fatigue forces at a frequency of 5 and 10 Hz.

	Average Static Breaking Force (N)	70% of an Average Static Breaking Force (N)	80% of an Average Static Breaking Force (N)	90% of an Average Static Breaking Force (N)
Frequency of 5 Hz	551	385	441	496
Frequency of 10 Hz	591	414	473	532

**Table 3:** Display of average density values, compression fatigue forces, number of cycles achieved and modulus of elasticity (E) at frequency of 5 Hz.

Frequency (Hz)	Percentage of Static Breaking Force (%)	Density (g/cm^3^)	Fatigue Force (N)	Number of Cycles	Modulus of Elasticity E (MPa)
5	70	0.451	385	2,040,364	9.711
5	80	0.454	441	743,615	9.787
5	90	0.463	496	296,451	10.163

**Table 4:** Display of average density values, compression fatigue forces, number of cycles achieved and modulus of elasticity (E) at frequency of 10 Hz.

Frequency (Hz)	Percentage of Static Breaking Force (%)	Density (g/cm^3^)	Fatigue Force (N)	Number of Cycles	Modulus of Elasticity E (MPa)
10	70	0.474	414	2,397,888	11.067
10	80	0.480	473	557,281	11.067
10	90	0.480	532	22,099	11.128

## Data Availability

The original contributions presented in the study are included in the article, further inquiries can be directed to the corresponding author.
